# Extra Supplement—The Nursing Mirror

**Published:** 1890-06-07

**Authors:** 


### "The Hospital" Nursing Mirror.

Being the Extra Nursing Supplement of "The Hospital" Newspaper.

All Contributions for this Supplement should be addressed to the Editor, The Hospital, 140, Strand, London, W.C., and should have the word "Nursing" plainly written in left-hand top corner of the envelope.

### En Passant.

ZENANA MISSION AT LUCKNOW.—The report of this medical mission for 1889 has just reached us, and tells what good work women are doing in India. The staff at Lucknow consists of Miss Mead, L.R.C.P., and Miss Haskew, M.D., as medical officers; Mrs. Thomas, as matron; three native Christian nurses, a male dispenser, and a female attendant for the out-patients. The surgical work seems to be specially successful—35 major operations and 21 operations in general surgery (including four abdominal sections), and no fatal case. One gratifying circumstance is that 45 of the women treated were *pardah-nishin*, and therefore could only have been treated by women.

THE OLDEST NURSING INSTITUTION.—An interesting gathering was held on Thursday, May 8, at 4, Devonshire Square, Bishopsgate, to celebrate the fiftieth anniversary of the Institution of Nursing Sisters. It was founded by Mrs. Fry and Lady Inglis, under the auspices of Queen Adelaide, by whose request the name of Nursing Sisters was given. The institution now consists of nearly a hundred nurses, who are in constant employment. A large number of nurses, probationers, and pensioners assembled at the invitation of the committee, and after tea the Marchioness of Tavistock (president of the institution) addressed them upon the special duties, difficulties, and privileges of a nurse's life. A silver monogram brooch, designed for the occasion, was presented to each of the nurses.

A HOME OF REST FOR NURSES.—This title certainly is rather suggestive of the Dogs' Home at Battersea, and of the Home of Rest for Horses; still we cannot print all the strictures on the scheme which have been sent us. One cutting which reaches us from the *Manchester Weekly Times* puts the matter succinctly:—"Nothing was more humiliating for the nurses present at the meeting than to hear the way in which their poverty was spoken about, and to have to listen while charity was begged for them. It is a sad fact that trained nurses are underpaid, but the remedy lies in going to the root of the matter—in teaching the nurses to combine together to raise the status and the pay of their noble profession, and not in building a charity home for them, as though they belonged to the idle or incapable classes."

A NOTE FROM THE NORTH.—The Ayr Association for providing trained nursing for the sick poor, issues its fifteenth annual report. The nurse, Miss M'Callum, has been most assiduous, and has always been welcomed by the patients whom she has visited. She has paid 4,310 visits to 296 patients, of whom 75 were men, 164 women, and 57 children. There were also 80 casual patients, of whom 25 were men, 39 women, and 16 children. 41 patients, of whom 20 were men and 21 women, were sent to the Ochiltree Convalescent Home, and 4 children to the Dundonald Convalescent Home. The balance-sheet shows the income for the year to have been as follows:—Balance from last Account, £170 18s.; Subscriptions for 1888-89, as per Subscription List, etc., £203 9s. Sums received for special cases, £25 3s. 6d. Interest from British Linen Co.'s Bank, £1 1s. 8d. Total £400 12s. 2d. After meeting all the expenses of the year a balance remained at the credit of the Association of £199 5s. 9½d.

HYPNOTISM.—The popular excitement about hypnotism is spreading to nurses, and we wish to warn our readers to be very careful how they speak about this difficult subject. We know of one nurse who lost a good connection amongst medical men by trying to practice this art, though her first efforts were encouraged by an enthusiastic young practitioner. The lecture lately delivered on Hypnotism at the Nurses' Club in Buckingham Street was excellent, and a summary of it will be found in *Nursing Notes* for June.

A NURSE AND HER HUSBAND.—A correspondent sends us an advertisement for a married couple for the Halifax Borough Fever Hospital; the wife to be a certificated nurse and manage the hospital, the husband to act as porter, clean windows, carry coal, "and perform any other duty generally desired by the committee or the matron." This is the supremacy of woman with a vengeance! A certificated nurse is generally a lady, must at least be a woman of fair education and intelligence, and is not likely to marry a man who would condescend to be her servant—in public, at least. Halifax is said to be 50 years behind the times, we think it is 100. Certainly the Committee will never get a good matron while their present rules stand.

SHORT ITEMS.—Last Saturday's *Queen* contains a portrait and notice of Miss Luckes, matron of the London Hospital. Strangely enough, on the same page appears a portrait and notice of Miss Margaret Harkness, a frequent visitor to the London, and for a short time a worker at Guy's.—"Cyril Bennett" is the nom-de-plume of Miss Rose Ingleby, who wrote the "Massage Case."—A correspondent writes: "I see a paragraph in the *British Medical Journal* headed 'Registration or Muzzling.' I haven't time to read it, but being a nurse and worried to death with the perpetual talk of registration, I throw in my vote for muzzling. If one or the other is necessary to prevent nurses inoculating their patients with bad views, I think that muzzling will be the least trying and irritating of the two."

NEWARK NEWS.—Some difficulty has arisen at Newark with regard to the district nurse, who at present works under the Hospital Committee, but who will probably in future be supported by special voluntary contributions. A writer to the *Newark Herald* says: "I move amongst the poor a great deal, and am constantly brought in close contact with cases which are, or have been, greatly relieved by the kind attention and persistent perseverance of Nurse Evans. I have no wish to enter into the question as to the authorities taking the steps they have done, with regard to discontinuing the services of Nurse Evans; they have a perfect right to do so if they think well. But everyone must acknowledge the fact that the way they have proceeded to carry out their designs is most unbusinesslike, and also calculated to damage the reputation and future usefulness of the nurse. The managers also seem to be quite unaware of the fact that the moral sentiment of the town is shocked at their action, which is seriously at variance with their opinions; and if the managers persist in carrying out their decision the whole town will be moved from end to end. I hope the question contemplated of opening a public subscription will be proceeded with at once; and also of taking the management out of the hands of the Hospital authorities."

### The British Nurses' Association and its Solicitor.

Our readers will remember that in the Nursing Supplement of The Hospital of May 25th, 1889, page xxviii., the following communication from Mr. F. S. Randolph, the solicitor to the B.N.A. appeared :—

THE BRITISH NURSES' ASSOCIATION AND THE PRESS.

The following extraordinary communication was sent by hand to our office at the beginning of the week :—

"3, Old Serjeants' Inn, Chancery Lane, E.C.,
" May 20, 1889.

"British Nurses' Association.

" Sir,—My attention has been called to an article at page xxv. of the Nursing Supplement to The Hospital of 18th May, reflecting upon the above association. As my instructions are to take immediate proceedings for libel whenever it may be necessary to do so on behalf of the association, I shall be obliged by your furnishing me with the name and address of "A Hospital Certificated Nurse" forthwith, that I may lay the matter before the executive committee of the association, and advise them thereon.—Yours obediently, "F. S. Randolph.

We stated at the time that we considered the letter complained of to be only fair criticism on a matter of public interest. We, therefore, declined to give up the name of the writer, and referred Mr. Randolph to our solicitors, Messrs. Slaughter and May, 18, Austinfriars, E.C., and nothing further transpired.

Another Letter.

On the 4th inst. we received the following further literary effort from Mr. Randolph, which is both interesting and instructive, especially to those acquainted with all the facts. The production is so excellent an example of Mr. Randolph's style that we readily comply with his request to quote it in its entirety :—

3, Old Serjeants' Inn, Chancery Lane, London, W.C.
4th June, 1890.

Sir,—My attention has just been called to a grossly untrue and malicious attack upon the British Nurses' Association in your issue of May 24th in the paragraph headed "One of the Elite." The Miss Gertrude Johnstone, who was matron of the Teignmouth Infirmary, is not, and has never been, a member of the Association, nor has she applied for registration; consequently, the whole of your laboured indictment of the Association tumbles to pieces, and the malice which prompted it recoils on yourself. You have maligned the character of Miss Gertrude W. Johnstone, who is a member of the Association, without taking the slightest trouble to identify her with the late matron of the Teignmouth Infirmary. You make other equally malicious misstatements, but, like the scores of other falsehoods you have printed concerning the Association and its members, these are deemed beneath notice or contempt. If, however, this letter in its entirety does not appear in this week's issue of The Hospital, I am instructed to inform you that steps will forthwith be taken to call public attention to the persistent malice exhibited in your periodical to the British Nurses' Association and its members.—I am, Sir, your obedient servant,
F. S. Randolph.

To the Editor of The Hospital, 140, Strand.

We did not nor have we ever mentioned Miss Gertrude W. Johnstone. In these circumstances we shall be curious to learn from Mr. Randolph how he justifies the statement that the paragraph headed " One of the Elite " " is a grossly untrue and malicious attack." May we further inquire when a *corrected* and full list containing the names, addresses, and qualifications of the nurses on the Register of the B.N.A. will be published? Until such a list is forthcoming, neither nurses nor the public can judge how far it may be wise or possible to countenance this Register, or to condemn it on its merits.

### The Joys of Johannesberg.

The following extract from a letter from Miss Rose Blennerhassett, has been forwarded to us for publication. Miss Blennerhassett, left a post of head nurse where she was highly valued to go to Johannesberg, because it was represented to her that nurses were badly needed there. She finds, as her lively notes show, a state of affairs it is well other nurses, who perhaps can less afford such experiences, had better note :—

May 19th—Our servants have run away—only a Kaffir boy remains for the work in the house and the house next door, where there are nine typhoid cases. Miss Kirkpatrick (late of Charing Cross Hospital) acts as home Sister, helped by Miss Hickman (late Sister at Carmarthen Hospital). These two and the "pro." have to do all the work of the home to day sweeping, cleaning, &c. This is our day.

Miss Mollett and I are busy here doing the dinners. I hear Miss M. spouting Schiller in the dining-room, whilst she is doing the grate out and lighting a fire. I am in the kitchen washing the breakfast things and trying to make the sleepy Kaffir boy clean saucepans. I set up an opposition to Schiller, and begin "Great wits to madness nearly are allied, And slight division do their bounds divide." I wave a spoon at the Kaffir—called Cornelius Agrippa. He drops his saucepan and disappears into a sort of packing-case house in the garden, where he lives; there he lies down for half an hour to compose his nerves. I give up Shakespeare and turn out an awful pantry: Miss Mollett comes and sets to work on the dinner. She has to cook it entirely for us, and for the convalescent patients next door, who have the ravenous, yet fastidious, convalescent appetite. We have great fun and laughing over the cleaning and the dinner, and finally some very good mince and soup is produced for the patients, and some uneatable fried fish for us; but, luckily, there is plenty of bread to-day and some Dutch cheese, so we needn't starve.

Here is a boy on horseback; it is the butcher's boy come again for his little cheque. He comes every day. I go and explain that we have no money. He is told so every day, and he always comes back looking quite hopeful. I tell him he'll be paid the week of the four Thursdays, and he rides off grinning from ear to ear.

For this home is really a dreadful ——? The late English clergyman here, doesn't seem to have understood accounts. I'm sure he did his best, poor thing; but since he left there has been a row about the church affairs; and, as regards this home, he certainly conveyed the impression that there was plenty of money, and that Miss Mollett would be able to make this a nursing centre, have a hospital, etc., whereas there is an enormous debt; and on arriving here Miss Mollett found £5 in the bank *pour tout potage*. She is a charming woman, with delightful manners, very well bred and unusually cultured. Just the person to be at the head of Bart's, or scme big London hospital, and just the last person who ought to be here cooking and cleaning. The people are extremely good, and help all they can, but there is no money in the place. The "Golden City" is bankrupt; people leave it in shoals every day. Without money it is only possible just to scramble along and look after the few young fellows who can be admitted to the Home. We have eleven beds, and a couple of wards have been run up at the back, which will be opened shortly. The people are getting up a subscription ball for the Home, so the butcher's boy has a chance! The young fellows we take in are supposed to pay £5 a-week, stimulants, doctor's bills, and drugs extra; they can't always pay, poor lads, and it seems a good deal to ask of them, and yet, what can one do in a place where eggs are 6s. a dozen, milk 6d. a pint, and everything else dear in proportion. *Apropos* of drugs, I am so bad, that I have wondered whether the doctors are in partnership with the chemists! You never saw anything to compare with the patients' prescription boards. They really are curiosities of literature, and one wonders that any enteric case, swallowing such a quantity of horrible stuff, and changing his medicine nearly every day, should ever survive. But some of them do recover, in spite of the treatment; about 20 per cent. die.

Miss Sleeman, from Guy's, the nurse who came out with me, is nursing a fever case a little way out of Johannesberg, but the epidemic is disappearing, and so, I hear, are a good many "bars"—the two facts probably have some connection!

The most intolerable thing here is the dirt. There is a thick, sticky, red dust everywhere. If you walk out, you come in coated with it, and there is very little water and considerable difficulty about washing. Our home looks out on "Government Square," a sort of square of dust and mud, with zinc shanties scattered irregularly over it.

No letters are delivered here. We have a box at the post office with a key, and we go and fetch them ourselves. It is very curious to see the post office pigeon-holed to the ceiling with these boxes. On Monday afternoons, when the English mail comes in, you may imagine how crowded the place is. I will not tell you my impression of Johannesberg, or whether I think I was wise or foolish in coming here. You shall draw your own conclusions from the above facts, and tell me what they are.

### Nurse Joyce.

(*Concluded from page xxxi.*)

Part II.

"Why don't you finish your breakfast, Oliver?" asked Anne when her own was over. Receiving no answer, she went on: "What shall you do about Lily?"

"Go up to London," said Oliver abruptly.

"Going up to London!" called out a cheery voice, and its owner, pushing open the door, entered the room; somebody, evidently, with the "freedom of the city" or rather the parsonage.

There were gossips who opined that Walter Beamish, of Glyn Fell, would willingly have carried off as mistress of the great farm of the district one of the parsonage sisters; which, however, they could not quite decide. But seeing that Anne was still to be had for the asking, as they amiably put it, the balance inclined towards the absent Lily.

"What in the world should take you to London, old man?" went on the welcome intruder, seating himself at the breakfast-table, and taking the cup of tea Anne offered him.

Walter Beamish was one of the oldest friends the Joyces had, and, in a few words, Oliver told him the circumstances of Lily's serious illness, and his own anxiety about her. But the only answer the stalwart young farmer made was to let his clenched hand fall resoundingly upon the table.

"I always said it would happen," Anne began to whimper, "but Lily never would listen. I was always against her going among those horrid people, with nasty, catching diseases. So forward of her, too—so unwomanly!"

The blood rushed to the visitor's brow, swelling the veins out like cords, and he steadily regarded the pale, placid woman at the head of the table.

"I have yet to learn," he spoke in harsh, grating accents, his breath coming and going quickly, "that it is unwomanly to tend the sick and ailing. We men-folk have a way of thinking otherwise, Miss Anne."

A slight colour tinged Anne's face at the sternly uttered words, and she discreetly kept silent. Anne had a capacity for silence, to give her what was her due; we each have some one redeeming point, possibly.

"Oliver"—Walter pushed away his tea to lean earnestly across the table—"let me run up to London. I've more time than yourself. I could take rooms near the hospital, and be on guard, as it were, for any change in—your sister; and I could wire down to you. Let me, old man; you know we've been like brothers." There was another reason Walter could have urged. His funds were ample; running up to London meant nothing at all to him, while with the country parson it would be otherwise.

"You!" The Rev. Oliver stared, "and why you?"

Walter flushed, and was silent. Then, with a great effort, he spoke: "Why, do you ask? Well, this is why, man to man" (he unconsciously ignored Anne's presence for the moment). "Lily is dearer to me than any woman upon earth; dearer than my own life, which I'd give freely to save hers. Perhaps I should not tell you this, as it seems she has not done so; but Lily refused to be my wife before she set out on the career she firmly believed to be her duty."

There was a stillness in the room as Walter ceased speaking. The Rev. Oliver pushed up his spectacles, and gazed, in dumb surprise, at his friend. And, in that silence, a certain ship went down. Into Anne's heart crept a dull despair—a despair that told her how firmly rooted had been the secret hope of Walter Beamish's love being offered for her own calm acceptance. It was the supreme moment of Anne Joyce's existence; let us not pry into its workings, they have their heart-hurricanes, these passionless women.

It all ended in both the Rev. Oliver and Walter going up to London, arriving in time to hear the good news that Nurse Joyce was out of danger—the tension of suspense was over.

"She shall come home to me," said a visiting physician who had known her father in the old days at King's, and he took the lily—now weak and fragile enough for all poetical purposes—to his West End home, where his girls and his good-natured wife vied with each other in nursing the invalid.

Then came a day for the tedious journey down to the North. The two men, with tenderest care, brought back Lily to her home. "The breezes of the hills will set her up as nothing else could," had been the medical verdict; "but she will never nurse anyone again," was added *sotto voce.*

"So you've got home again, Lily," said Anne, quietly. Perhaps she was half-melted at the sight of the sweet, white face. Still, there rankled in her heart the old grudge against the career Lily had so determinedly undertaken, and, possibly, some other bitter feeling as well.

"Yes, Anne. There's no place like home when there's anything the matter with us," and Lily laughed gently, the sweetest music on earth in somebody's ears.

They were all sitting, in the twilight, in the quaint parsonage sitting-room, and Lily was lying on the old-fashioned sofa, looking lovingly at the dear old hills. It seemed very good to be still living, and the brooding sense of thankfulness belonging to convalescence was in the girl's heart.

"Lily," said Anne, presently, "tell me, for I don't know yet, how it was you got this horrid illness. For my part, I can't see why there was a necessity for you to get your blood poisoned."

"Ah, but Anne, if you had known my little patients! There was a wee, motherless child whom they brought into hospital. How we watched over the blue-eyed mite! And she took a liking to me; she would have me always with her if she could. Wasn't it strange?" Somebody stirred quickly in the half-light; it was Walter Beamish; perhaps he didn't think the fact such a strange one. "I was so tired, exhausted, you know. One of the sisters used to help me with her, but she wailed for me: 'Mammy! I want my Mammy-nurse!' She always called me so; perhaps I may have reminded her of the mother she had recently lost. I was constantly sent for to pacify the child, and the tiny arms went tight—so tight—round me and—"

"And" interrupted Anne roused to eagerness, "you didn t shake off the child, and run away as I should have done?"

"Run away!" Lily was bewildered. As well might Anne have asked a soldier if he turned, and ran when the first bullets whizzed round his head. But, then, Lily was one of the women whom Anne deemed unwomanly.

"Well?" persisted Anne, "and wouldn't that have been the wisest course, when you positively knew you were breaking down?"

There was a pained silence. Then Oliver rose, slowly crossed the room to the old sofa, and laying his hand on the fair head resting there, he said reverently, "God bless you, child, you did your duty. And, Anne," turning to his elder sister, "there will, of a certainty, be a day when our sister will listen to the blessed words, 'Inasmuch as ye have done it unto one of the least of these My brethren, ye have done it unto Me.' And they will refer to that little one, to comfort and support whom Lily risked her own life."

Nurse Joyce is still remembered lovingly in her hospital, but she is only a memory there, not a personality, for Lily never regained the amount of strength necessary to her beloved profession, and without it she had sufficient wisdom to know her uselessness. But she has, at least, the intense satisfaction of knowing that "she hath done what she could."

And she is not a cumberer of the ground, an aimless woman, by any means, for she is the light of a good man's eyes, the eyes of her husband. Lily is mistress of Glyn Fell, at last, and "looketh well to the ways of her household."

So, after all, Anne Joyce has got her will, Lily's career as a worker in the great world has abruptly ceased, but—ah, there is always a but, and, perhaps, to Anne it is a bitter one

### Everybody's Opinion.

### RURAL NURSING ASSOCIATION.

"A MEMBER OF THE COMMITTEE" writes: May I ask you to be so good as to correct a paragraph in your issue of the 17th inst. concerning the Kemerton District of the above association? We are glad to say there is no change in our kind and excellent secretary, and all our fellow-workers are *entirely* in favour of thoroughly-trained nurses. We are quite satisfied with our present nurse. As the Committee comprises a fair proportion of gentlemen the ladies can give their care more especially to the comfort of the sick poor, who are unfeignedly grateful for the help afforded.

### HOLIDAY HOMES.

A. E. S. writes: I am so glad there is a feeling in THE HOSPITAL of May 24th that "a special home" is not the need for nurses, but a special fund from which each hospital, on proper application, could be supplied with a certain sum in proportion to the number of nurses, so that life could be made brighter in the summer by drives and sails, and in the winter by a few concert tickets. Most of us can find money for our one holiday and a friend to take us in, even if we have out-lived our "home ties." But we do begrudge money for pure amusement; the consequence is, we seldom go out of the beaten walk. Few people ever give a thought to the tired nurse. We are "made to go," and are not expected to be tired, worn out, or suffer in any way; and hospitals are so under-nursed that it is difficult to find an extra nurse for a special case or for an operation without putting more work on the all too-tired shoulders. Matrons are often blamed for over-working nurses, but without reason. She is always in fear the expenses will run up if she has any help, and the public will not give; and the doctors, too, so often thoughtless and unpunctual, keep a nurse waiting whilst they smoke a pipe or chatter with a friend. Poor nurse cannot say a word, but her off-time is shortened, and cannot be picked up again. "Want of thought, not want of heart," is the fault.

"ANT" writes: I am obliged to say just a word about nurses' holidays. I saw in the *Daily News* some days ago that ladies were discussing a home of rest for nurses. If those in authority would only see that nurses got longer holidays, the majority of them would soon enough find places to go to. For instance, at Guy's, nurses get two weeks in the year, unless it has been altered lately, and as some live more than a day's journey from London, the travelling takes rather a slice from their holiday. If they could only have a fortnight every six months, or at their own option a month every year, I am sure it would go far towards keeping them in good health. I believe the above is the plan adopted at the Evelina. Private nurses are much better off in that respect, and if, like myself, working alone, can take a holiday when they need it, always, of course, funds allowing. But we do not want to go to a large house altogether, which would only be another form of institution life without the work. Will you kindly forward me a photograph of Mr. Junius Morgan, to whom nurses can never be grateful enough?

### Death in our Ranks.

WE regret to record the death of Miss Haynes, Lady Superintendent of the General Hospital, Barbadoes. Miss Haynes received her training and certificate at the Royal Free Hospital, and died of pneumonia within a year of her appointment.

### Lecture.

THE next Lecture at the Midwives' Institute and Trained Nurses' Club will be given on Friday evening, June 20, at 7.45, by Dr. Leith Napier, F.R.S., Ed. Subject:—"The Thermometer in Gynæcology (Second part). A few tickets to non-members 6d. each.

### Notes and Queries.

### Queries.

(15) *Home Wanted.*—Would some of our readers kindly give any information they can about homes for epileptic girls, the North of England preferred?—*E. H.*

(16) *Private Cases Wanted.*—Can anyone tell me of an institution or home where non-resident nurses are supplied with cases?—*A. C.*

(17) *Knitted Purses.*—Will some one give instructions for making these purses?—*L. W.*

(18) *Encyclopædia.*—Can anyone tell me where to get a good second-hand Encyclopædia?—*India.*

### Answers.

*Miss R. S.*—The following is the paragraph to which you refer. It appeared in THE HOSPITAL for March 2nd, 1889: "Nurses often complain of swollen feet, especially when they first go into hospital. A powder which is much used in the German army for sifting into the shoes and stockings of the foot soldiers might be of service to them. It consists of three parts of salicylic acid, ten parts of starch, and eighty-seven parts of pulverized soapstone. This keeps the feet dry, prevents chafing, and heals any sore spots. The soapstone alone, without the other ingredients, has also been found useful."

*A. M. S.*—We never prescribe; consult a medical man.

*Judy.*—(1) The Cancer Hospital, Brompton, S.W. (2) Always address the house surgeon as "Sir."

*** Correspondents whose communications do not appear in the current number are requested to refer to the following week's issue.

### Vacancies.

THE following vacancies are announced:

**Matron.**

Woolwich Cottage Hospital; £40.

**Night Superintendent.**

Bristol Royal Infirmary; £40.

**Sister.**

Devonport Royal Albert Hospital.

**Nurses.**

Berry Wood Asylum, Northampton; £20.
Birkenhead Borough Hospital; £25.
Bishop's Stortford Home; £25.
Blackburn Infirmary; £25.
Blackheath Institution.
Brighton Children's Hospital; £20.
Burton-on-Trent Nursing Institution.
Canterbury Nurses' Institute.
Cheltenham General Hospital.
Chester Deaconess' Home; £24.
Clapham and Brixton Nursing Institution.
Clayton Hospital, Wakefield; £25.
Frome Nurses' Home; £25.
General Nursing Institute.
Huddersfield Nurses' Home.
Leeds Nurses' Institution; £25.
Levick Institution, Paris.
Liverpool Nurses' Institution.
Maghull Home; £20.
North-West London Hospital; £24
Ophthalmic School, Hanwell; £26.
Oldham Workhouse Infirmary; £30.
Shoreditch Infirmary (night); £20.
St. Helena Home, N.W.
St. Olave's Union Infirmary; £25.
Surbiton Nurses' Institution.
Walsall Cottage Hospital, £18.
Western Fever Hospital, S.W.; £30.
Weston-super-Mare Institute.
Wirral Children's Hospital.
Woolwich Cottage Hospital; £20.
15, Fitzroy Square.

**District Nurses.**

Canterbury Nurses' Institute.
East London Nursing Society.
Middlesboro' Nurses' Home.
St. Andrew's, Plymouth.

**Probationers.**

Bath Nurses' Home.
Bolton Infirmary.
Clapham and Brixton Nursing Institution.
Clayton Hospital, Wakefield; £12.
Cromwell House, Highgate.
Grimsby Hospital.
Hertford Infirmary; £12.
Hospital for Diseases of the Chest, E.C.; £12.
Lynn Hospital.
North-West London Hospital; £8.
Weston-super-Mare Hospital.

### Appointments.

BARBADOES GENERAL HOSPITAL.—Miss F. A. Marquardt has been appointed Lady Superintendent of this hospital. She holds the "Royal Free" Nurses' Certificate, where she trained for four years.

LADY STRANGFORD'S HOSPITAL.—Miss Nixon and Miss Nina Smalle Ryalls, both of the Children's Hospital, Great Ormond Street, have been appointed head nurses of this hospital in Egypt.

LOWESTOFT HOSPITAL.—Miss E. A. Wildman, of the Royal Hants County Hospital, Winchester, has been appointed matron of Lowestoft Hospital, Suffolk.

MONSALL FEVER HOSPITAL, MANCHESTER.—Miss A. Pratt has been appointed Sister of this hospital. Miss Pratt trained for four years at the Royal Free Hospital, Gray's Inn Road, and for the last twelve months has had charge of one of the medical wards there.

### Amusements and Relaxation.

**N.B.—New Word Competition Commenced April 5th, 1890, ends June 28th, 1890.**

Proper names, abbreviations, foreign words, words of less than four letters, and repetitions are barred; plurals, and past and present participles of verbs, are allowed. Nuttall's *Standard* dictionary only to be used.

The word for dissection for this, the TENTH week of the quarter, being

"COACHING."

| Names. | May 29th. | Totals. | Names. | May 29th. | Totals. |
|---|---|---|---|---|---|
| Adeline | — | 149 | Agamemnon | 85 | 352 |
| Patience | 80 | 353 | G. E. J. C. | — | 34 |
| Lightowlers | 78 | 335 | S. E. A. | — | 24 |
| Canary | — | 86 | Jenny Wren | 72 | 327 |
| M. E. S. | — | 37 | Multum in parvo | — | 32 |
| Ambition | — | 36 | Ecila | — | 39 |
| M. W. | — | — | Weta | 55 | 259 |
| Esperance | — | — | Henri | — | 161 |
| Judy | — | 37 | Holland | 82 | 351 |
| Reldas | 81 | 351 | T. J. | — | 111 |
| Merenda | — | 29 | Nurse Isabel | — | 17 |
| Lucy Locket | — | 40 | Nurse Faith | — | 9 |
| Camellia | — | 30 | E. S. Z. | — | 10 |
| Qu'appelle | 78 | 339 | G. P. | — | 10 |
| Tinie | 80 | 353 | Stumps | — | 33 |
| Nurse Mildred | — | 190 | Bolton | — | 8 |
| Coralie | 84 | 349 | Jesmur | — | 53 |
| Gladys | 79 | 348 | Ran | — | 17 |

